# Serum chemokine (CC motif) ligand 2 level as a diagnostic, predictive, and prognostic biomarker for prostate cancer

**DOI:** 10.18632/oncotarget.6690

**Published:** 2015-12-19

**Authors:** Kouji Izumi, Atsushi Mizokami, Hsiu-Ping Lin, Hui-Min Ho, Hiroaki Iwamoto, Aerken Maolake, Ariunbold Natsagdorj, Yasuhide Kitagawa, Yoshifumi Kadono, Hiroshi Miyamoto, Chiung-Kuei Huang, Mikio Namiki, Wen-Jye Lin

**Affiliations:** ^1^ Department of Integrative Cancer Therapy and Urology, Kanazawa University Graduate School of Medical Science, Kanazawa, Japan; ^2^ Immunology Research Center, National Health Research Institutes, Zhunan, Miaoli County, Taiwan; ^3^ Departments of Pathology and Urology, Johns Hopkins University School of Medicine, Baltimore, MD, USA; ^4^ Department of Medicine, The Warren Alpert Medical School of Brown University, Providence, RI, USA

**Keywords:** androgen deprivation therapy, biomarker, CCL2, prostate cancer, risk classification

## Abstract

Prostate-specific antigen (PSA) is regarded as the most sensitive biomarker for prostate cancer. Although androgen/androgen receptor (AR) signaling promotes prostate cancer progression, suppression of AR signaling induces chemokine (CC motif) ligand 2 (CCL2), which enables prostate cancer cells to gain metastatic potential. AR-controlled PSA alone may be an unreliable biomarker for patients receiving androgen deprivation therapy. Therefore, we investigated the validity of CCL2 as a complementary biomarker to PSA for prostate cancer. Our *in vitro* approach of enriching for prostate cancer cells with higher migration potential showed that CCL2 activated cellular migration. Importantly, we found that CCL2 levels were significantly different between men (*n* = 379) with and without prostate cancer. Patients with CCL2 ≥ 320 pg/mL had worse overall survival and prostate cancer -specific survival than those with CCL2 < 320 pg/mL. A novel risk classification was developed according to the risk factors CCL2 ≥ 320 pg/mL and PSA ≥ 100 ng/mL, and scores of 2, 1, and 0 were defined as poor, intermediate, and good risk, respectively, and clearly distinguished patient outcomes. CCL2 may serve as a novel biomarker for prostate cancer. The novel risk classification based on combining CCL2 and PSA is more reliable than using either alone.

## INTRODUCTION

Prostate cancer is the most common malignancy and the second leading cause of cancer death in males in the United States [1]. Because androgen/androgen receptor (AR) signaling promotes prostate cancer progression, standard treatment for patients with advanced prostate cancer employs androgen-deprivation therapy (ADT) [2-4]. However, prostate cancer often progresses to castration-resistant prostate cancer (CRPC) after several years of ADT [5]. Although prostate-specific antigen (PSA) is a reliable biomarker for prostate cancer, it has significant limitations [6, 7]. We previously showed that the suppression of AR signaling not only inhibited prostate cancer cell proliferation and PSA secretion but also promoted CCL2 secretion, enabling prostate cancer cells to metastasize [8]. AR-controlled PSA alone lacks reliability as a biomarker, and identification of complementary biomarkers to PSA is required for accurate prediction. In the present study, we investigated the validity of chemokine (CC motif) ligand 2 (CCL2) as a biomarker complementary to PSA in prostate cancer diagnosis and prognosis.

## RESULTS

### CCL2 induces prostate cancer cell migration *in vitro*

**Figure 1:**
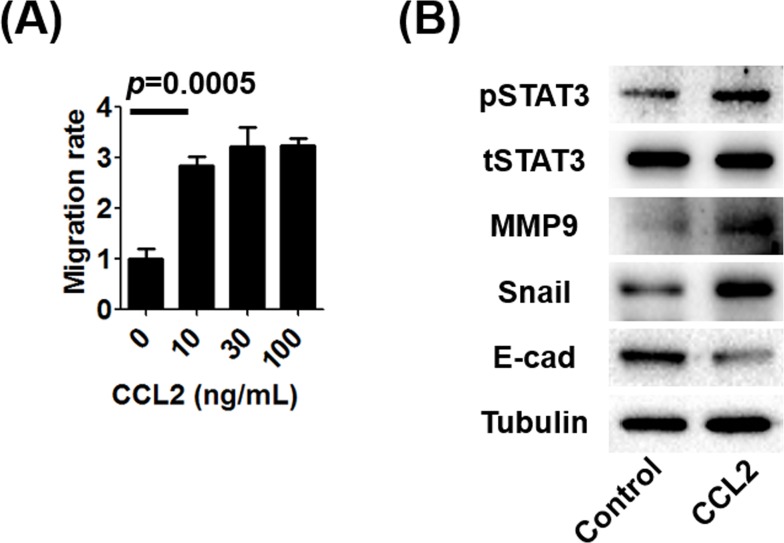
The effect of CCL2 on human prostate cancer C4-2 cells **A.** The migration of C4-2 cells in the absence of suppression of androgen/AR signaling was performed using 24-well transwell inserts and plates with or without treatment using the indicated concentrations of CCL2. **B.** WB analyses of mesenchymal markers (MMP9 and Snail), an epithelial marker (E-cadherin), and STAT3 in C4-2 cells after treatment with 10 ng/mL of CCL2 were performed and compared with the control. The activation of STAT3 (pSTAT3), which is a key molecule bridging between CCL2 and EMT, was associated with the EMT.

To determine the *in vitro* effects of CCL2 stimulation on prostate cancer cell migration without androgen deprivation, we used a transwell migration assay. We found that CCL2 induced prostate cancer cell migration (Figure 1A). Next, we analyzed the levels of markers that are often associated with enhancing cellular migration in CCL2-treated prostate cancer cells. Western blot (WB) analysis showed that CCL2 treatment upregulated mesenchymal markers such as Snail, MMP-9, and pSTAT3 and down-regulated the epithelial marker E-cadherin (Figure 1B).

### Prostate cancer cells with higher migration potential secrete higher levels of CCL2

**Figure 2:**
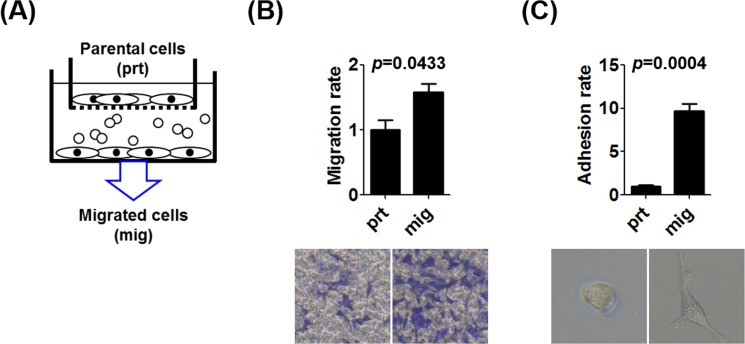
The enrichment of the population of prostate cancer cells with increased migration potential **A.** We established an *in vitro* transwell approach to select prostate cancer cells with increased migration ability. C4-2 cells that migrated from upper transwell inserts to the bottom of lower wells were called mig cells. **B.** The migration of C4-2 prt and mig cells were compared. **C.** C4-2 cells attached to the bottom surface after migration were counted, and the morphological change of mig cells into a polygonal shape was observed.

**Figure 3:**
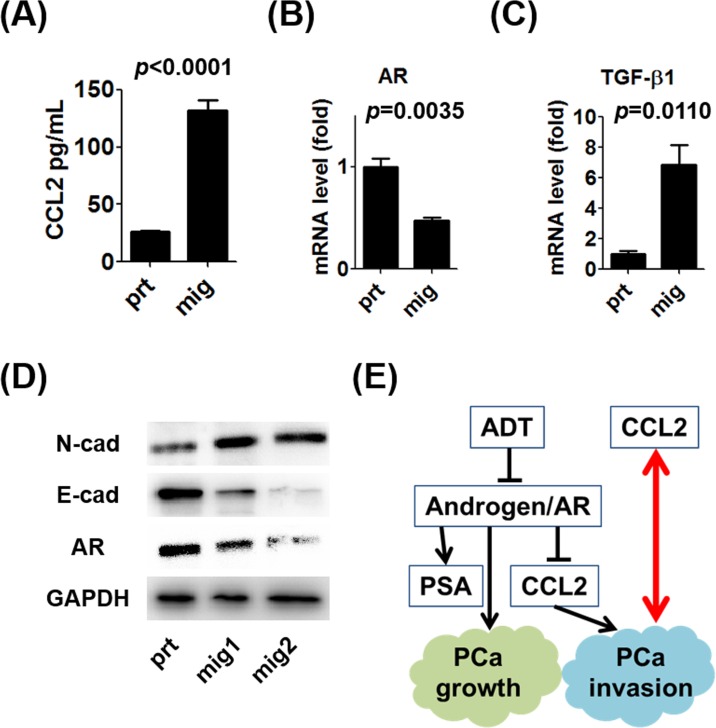
Characteristics of prostate cancer cells with increased migration potential **A.**CCL2 concentrations in supernatants of C4-2 prt and mig cells were measured using an ELISA kit. **B.** mRNA levels of AR expressed by C4-2 prt and mig cells were analyzed using qRT-PCR. **C.** mRNA levels of TGF-β1 in C4-2 prt and mig cells were analyzed using qRT-PCR. **D.** WB analyses of N-cadherin (mesenchymal marker), E-cadherin (epithelial marker), and AR expression by C4-2 mig cells were performed, and the results were compared with those for C4-2 prt cells (mig1 and mig2 were independently collected). **E.** These data indicate that CCL2 induced the migration of prostate cancer cells and the EMT *in vitro* in the presence or absence of the suppression of androgen/AR signaling and that prostate cancer cells with higher metastatic potential secreted more CCL2 (red arrow).

To enrich the population of prostate cancer cells with increased migration potential, we established an *in vitro* transwell model (Figure 2A). After selection, we confirmed that the ability of selected prostate cancer cells (mig cells) to migrate through the transwell and adhere to the lower chamber for growth (Figure 2B, 2C). The CCL2 ELISA data showed that autocrine CCL2 levels were significantly increased in the selected mig cells with increased *in vitro* migration ability (Figure 3A), implicating increased autocrine production of CCL2 by prostate cancer cells as a key step in the promotion of prostate cancer cell migration. We investigated whether the changes in the mRNA levels of two important contributors to the epithelial-mesenchymal transition (EMT), AR, and transforming growth factor-β1 (TGF-β1), were associated with increased autocrine CCL2 levels. In selected mig cells with increased CCL2 levels, the increased level of TGF-β1 mRNA correlated inversely with that of AR (Figure 3B, 3C), suggesting that during CCL2-mediated EMT, prostate cancer cells differentially regulated the levels of AR and TGF-β1 to enable prostate cancer cells to increase their ability to migrate *in vitro*. WB analysis showed an increase in the expression of the mesenchymal marker N-cadherin and a decrease in the expression of E-cadherin and AR (Figure 3D). These data suggest that autocrine production of CCL2 induced prostate cancer cell migration *in vitro* and that in the EMT, regardless of the presence or absence of the suppression of androgen/AR signaling, prostate cancer cells with higher migration potential secreted more CCL2 (Figure 3E). Therefore, CCL2 may serve as a biomarker for aggressive prostate cancer with higher migration ability.

### CCL2 as a diagnostic marker

**Figure 4:**
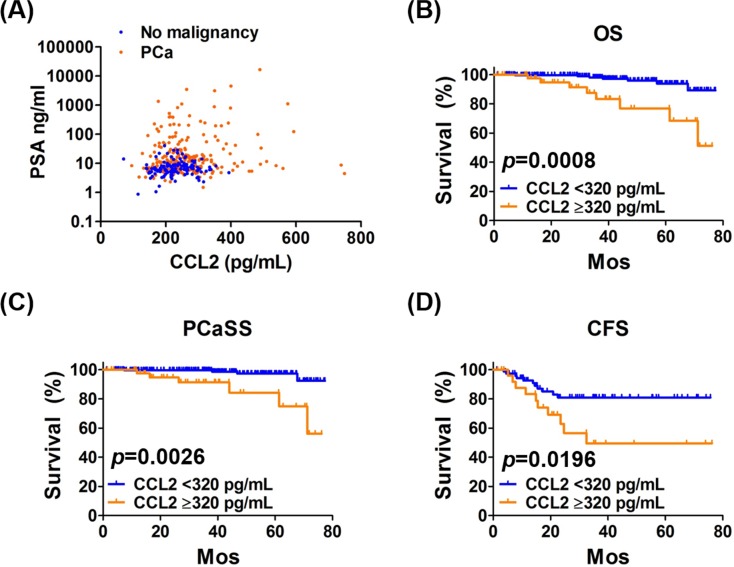
Analysis of PSA and CCL2 levels as functions of OS, PCaSS, and CFS **A.** The scatter plot of PSA (logarithmic scale) and CCL2 levels shows clustering of patients without prostate cancer around small areas of low PSA and CCL2 levels, and their levels in prostate cancer patients were widely scattered. PCa = prostate cancer. **B.**, **C.** Kaplan-Meier curves of OS and PCaSS in 255 patients with prostate cancer with CCL2 ≥ 320 pg/mL and CCL2 < 320 pg/mL. The 5-year OS rates of patients with CCL2 ≥ 320 pg/mL and CCL2 < 320 pg/mL were 76.9% and 93.9%, respectively. The 5-year PCaSS rates of patients with CCL2 ≥ 320 pg/mL and CCL2 < 320 pg/mL were 84.3% and 97.3%, respectively. **D.** Kaplan-Meier curves of the CRPC-free survival (CFS) of 102 patients treated with ADT and patients with CCL2 ≥ 320 pg/mL and < 320 pg/mL. The 5-year CFS rates of patients with CCL2 ≥ 320 pg/mL and < 320 pg/mL were 49.5% and 80.9%, respectively.

Next, we investigated the clinical relevance of increased CCL2 levels to support the findings of the *in vitro* experiments. Four potential serum biomarkers were measured in biopsies of 379 men who were screened before biopsy using the PSA test, digital rectal examination, and transrectal ultrasonography. There were 255 and 124 men with and without prostate cancer, respectively. The areas under the ROC curves for PSA and CCL2 were 0.722 and 0.609, respectively (Supplementary Figure S1). However, there was a statistically significant difference in CCL2 levels between men with and without prostate cancer (*p* < 0.0001) (Table 1). Scatter plots of PSA and CCL2 levels showed that men without prostate cancer clustered around a small area of low PSA and CCL2 concentrations, while those of patients with prostate cancer were widely scattered (Figure 4A). Because the highest PSA level of men without prostate cancer was 40.6 ng/mL, the levels of CCL2 in men with and without prostate cancer in specific PSA concentrations ranges were compared to determine if the CCL2 level was beneficial for assisting the diagnosis of prostate cancer of men with the lower PSA range. The level of CCL2 in patients with prostate cancer was significantly higher than those without prostate cancer in all PSA ranges ( < 41, < 20, < 10, 4-41, 4-20, and 4-10 ng/mL) (Supplementary Table S1). These differences in CCL2 levels between men with and without prostate cancer support the potential of serum CCL2 levels as a diagnostic biomarker for prostate cancer.

**Table 1:** Serum biomarkers in biopsied men

		No malignancy	PCa	*p*
*n*		124	255	
Median PSA, ng/mL		6.5 (0.9-40.6)	10.6 (1.5-16702)	0.1167
Median CCL2, pg/mL		224 (70.5-394)	246 (95.3-749)	<0.0001
Median TT, ng/mL		4.4 (0.0-10.7)	4.6 (1.4-11.7)	0.1867
Median TGF-β1, ng/mL		28.6 (8.3-48.8)	27.4 (8.8-48.4)	0.7947

PCa = prostate cancer, PSA = prostate-specific antigen, TT = total testosterone, TGF-β1 = transforming growth factor β1. Values in parentheses indicate range.

### CCL2 as a prognostic biomarker

**Figure 5:**
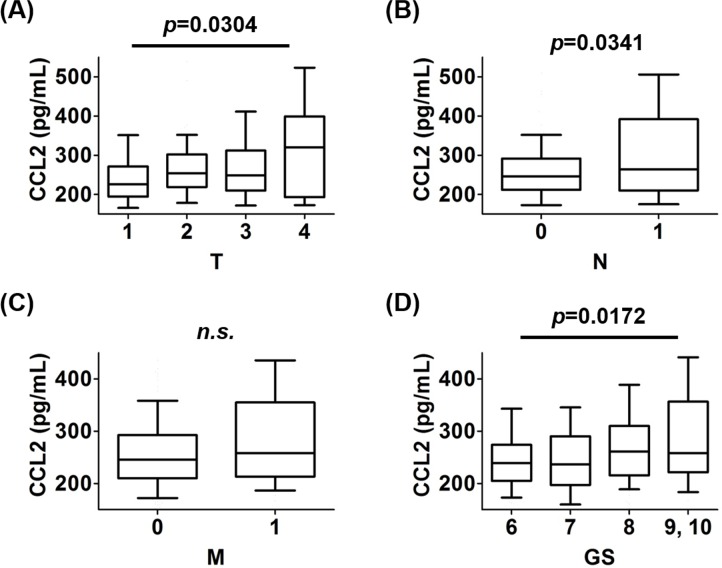
The prevalence of CCL2 values in each stage of TNM and GS **A.** The levels of CCL2 in patients with each T stage were analyzed. The level of CCL2 in patients with T4 was higher than those with T1. **B.** The levels of CCL2 in patients with each N stage were analyzed. The level of CCL2 in patients with N1 was higher than those with N0. **C.** The levels of CCL2 in patients with each M stage were analyzed. There was no significant difference in CCL2 levels between patients with M1 and M0 (*p* = 0.1277). **D.** The levels of CCL2 in patients with each GS were analyzed. The level of CCL2 in patients with GS ≥ 9 was higher than those with GS = 6.

To determine whether CCL2 served as a prognostic biomarker of prostate cancer, the levels of CCL2 were analyzed for their association with overall survival (OS) and prostate cancer-specific survival (PCaSS) in 255 patients. Patients with CCL2 ≥ 320 pg/mL had significantly poorer OS (*p* = 0.0008) (Figure 4B) and PCaSS (*p* = 0.0026) (Figure 4C) than those with CCL2 < 320 pg/mL. As shown in Supplementary Table S2, patients with CCL2 ≥ 320 pg/mL had higher TNM stages and Gleason scores (GS) than those with CCL2 < 320 pg/mL. However, there was not a significant difference in PSA levels at diagnosis or in treatment regimens between groups. When the prevalence of the CCL2 value was analyzed in each stage of TNM and GS, advanced stages showed higher CCL2 levels, except for M stage (Figure 5A-5D). However, the analysis of CCL2 levels revealed that patients with a higher CCL2 level had a higher prevalence of meastasis (Supplementary Figure S2).

### CCL2 as a predictive biomarker

Of the 255 patients, 102 underwent continuous ADT at the time of the latest follow-up, and their CCL2 concentrations ( ≥ 320 pg/mL) correlated significantly with poor OS (*p* = 0.0104) (Supplementary Figure S3A) and PCaSS (*p* = 0.0372) (Supplementary Figure S3B) compared with those with CCL2 < 320 pg/mL. Interestingly, patients with CCL2 ≥ 320 pg/mL (10/27, 37%) had a significantly higher risk of developing CRPC than those with CCL2 < 320 pg/mL (11/75, 15%) (*p* = 0.0196) (Figure 4D). These results indicate that CCL2 may serve as a biomarker that predicts the efficacy of ADT for prostate cancer.

### Novel risk classification combining CCL2 and PSA

**Figure 6:**
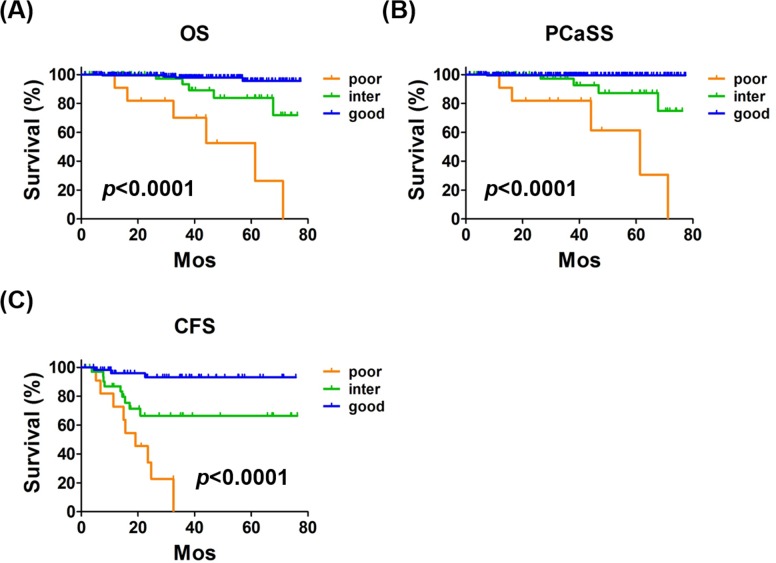
The novel risk classification The classification system was designed according to the number of risk factors (CCL2 ≥ 320 pg/mL and PSA ≥ 100 ng/mL) and 2, 1, and 0 were defined as poor, intermediate, and good risk, respectively. **A.**, **B.**) Kaplan*-*Meier curves of OS and PCaSS in 255 patients with prostate cancer with poor, intermediate, and good risk. The 5-year OS rates were 52.6%, 83.8%, and 95.6% of patients with poor, intermediate, and good risk, respectively. The 5-year PCaSS rates of patients with poor, intermediate, and good risk were 61.4%, 87.2%, and 99.5%, respectively. **C.** Kaplan-Meier curves of CFS of 102 patients with prostate cancer treated with ADT. The 5-year CFS rates of patients with poor, intermediate, and good risk were 0%, 66.5%, and 93.0%, respectively.

Consistent with the observations of a previous study [9], patients with PSA ≥ 100 ng/mL had significantly poorer OS and PCaSS than those with PSA < 100 ng/mL (Supplementary Figure S4A, S4B). Similarly, in patients treated with ADT, PSA > 100 ng/mL correlated with poor OS, PCaSS, and CRPC-free survival (CFS) (Supplementary Figure S5A-S5C). As shown in Supplementary Table S3, patients with PSA ≥ 100 ng/mL had higher TNM stages and GS than those with PSA < 100 ng/mL. Because CCL2 and PSA levels did not correlate, we reasoned that these two useful biomarkers might be a more powerful biomarker profile for prostate cancer when they were combined. The risk classification was generated according to the number of risk factors (CCL2 ≥ 320 pg/mL and PSA ≥ 100 ng/mL), and scores of 2, 1, and 0 were defined as poor, intermediate, and good risk, respectively. There were significant differences in OS (*p* < 0.0001) (Figure 6A) and PCaSS (*p* < 0.0001) (Figure 6B) overall among poor, intermediate, and good risk groups. There were significant differences in OS (*p* < 0.0001, Supplementary Figure S6A), PCaSS (*p* < 0.0001) (Supplementary Figure S6B), and CFS (*p* < 0.0001) (Figure 6C) among the patients from poor, intermediate, and good risk groups treated with ADT. As shown in Table 2, the risk classification divided prostate cancer patients into three groups that showed significant differences in TNM stages and GS. Moreover, this risk classification clearly distinguished each survival risk compared with the use of a single biomarker.

**Table 2:** PCa patients background according to risk

			poor	intermediate	good	*p*
Total, *n*			11	53	191	
Median age, yr			76 (52-79)	71 (46-86)	69 (50-89)	0.2205
T, *n*	1		1	6	54	0.0068/< 0.0001†
	2		1	23	108	
	3		3	18	26	
	4		6	6	3	
N, *n*	0		2	41	185	0.0004/< 0.0001†
	1		9	12	6	
M, *n*	0		3	41	186	0.0009/< 0.0001†
	1b		5	9	3	
	1c		3	3	2	
GS, *n*	6		0	7	52	0.0027/< 0.0001†
	7		0	15	83	
	8		2	17	34	
	9		8	10	19	
	10		1	4	3	
Treatment, *n*	cADT§		11	25	42	0.0032/0.0054†‡
		+HDR	0	4	7	
		+seed	0	1	3	
		+EBRT	0	4	3	
		+RP	0	0	2	
	tADT		0	0	10	
		+HDR	0	6	41	
		+seed	0	7	25	
		+EBRT	0	0	7	
		+RP	0	1	15	
	HDR		0	0	2	
	Seed		0	4	13	
	RP		0	1	16	
	AS		0	0	5	

PCa = prostate cancer, GS = Gleason score, cADT = continuous androgen-deprivation therapy, HDR = high-dose-rate brachytherapy, EBRT = external beam radiotherapy, tADT = temporary ADT, RP = radical prostatectomy, AS = active surveillance.

† Comparison poor and inermediate/comparison intermediate and good.

‡ Treatments are divided into 3 groups; ADT alone, ADT+local therapy, and local therapy alone.

§ One hundred were combined androgen blockade and 2 were monotherapy.

## DISCUSSION

Human CCL2 was identified in 1987 [10, 11]. Originally, CCL2 was reported as a chemical mediator attracting mononuclear cells to inflammatory tissue [11, 12]. Since Loberg et al. reported the detailed mechanism of prostate cancer progression *via* CCL2 [13, 14], the role of CCL2 in cancer progression was consistently shown in a variety of malignancies [15-17]. Initially, CCL2 was reported to promote prostate cancer metastasis through the recruitment of macrophages using PC-3 cells that did not express AR [18]. We further investigated molecular mechanisms by which AR inhibits CCL2 secretion in AR-positive human prostate cancer cell lines [8, 19]. CCL2 is secreted by prostate cancer cells and tumor-associated macrophages (TAM) during coculture conditions that mimic ADT and induces prostate cancer cell migration/invasion *via* CCL2-dependent STAT3 activation and the EMT pathways [8, 19]. Immunohistochemical analysis of tissue specimens suggests that patients with prostate cancer with high CCL2 expression in their tumors had worse OS and shorter time to recurrence after prostatectomy than those with low CCL2 expression [8]. Further, CCL2 induces resistance to anti-androgens *via* interaction between prostate cancer cells and TAM [8]. These results strongly support the potential of CCL2 as a biomarker of prostate cancer. However, it remains unclear whether CCL2 promotes prostate cancer progression in prostate cancer cells regardless of therapeutic targeting of androgen/AR signaling. Our present data indicate that CCL2 induces prostate cancer cell migration *in vitro* not only under ADT conditions but also without the suppression of androgen/AR signaling (Figure 1A, 1B). Furthermore, prostate cancer cells with higher migration potential secrete more CCL2 (Figure 3A). Therefore, CCL2 may serve as a biomarker from the time prostate cancer is diagnosed.

TGF-β1 is an EMT marker and promotes prostate cancer progression [20, 21] (Figure 3C). Higher serum TGF-β1 levels predict biochemical recurrence after prostatectomy [22]. Serum testosterone is a prognostic prostate cancer biomarker [23]. However, our present study shows a significant difference in CCL2 levels alone between men with and without prostate cancer. Interestingly, this result is consistent with a recent pilot study of potential biomarkers for prostate cancer showing that CCL2 alone may serve as a diagnostic serum biomarker among six chemokines [24]. Double screening using serum PSA and CCL2 may reduce unnecessary biopsies and facilitate more accurate diagnosis of prostate cancer.

Moreover, CCL2 may serve as a prognosticator for prostate cancer patients regardless of their disease status (Figure 4B, 4C). CCL2 may serve as well as a predictive biomarker for patients with prostate cancer treated with ADT (Figure 4D), suggesting that ADT alone might not be sufficient for patients with prostate cancer with higher CCL2 levels. Recent studies demonstrated the efficacy of combination therapy with ADT and cytotoxic chemotherapy as first-line treatment, particularly for selected high-risk patients [25]. Our present data support the conclusion that CCL2 status may help clinicians to select such patients. Interestingly, chemotherapy resistance mediated by CCL2 was reported, and inhibition of CCL2 activity was suggested to enhance therapeutic responses to taxane-based chemotherapy [26].

In addition to the absence of a strong correlation between PSA and CCL2 in patients with prostate cancer (Figure 4A), we demonstrated the limitation of PSA alone as a biomarker for prostate cancer [6, 7, 27]. Furthermore, mutually exclusive characteristics are observed between activation of androgen/AR signaling and CCL2 secretion [8]. Therefore, it is reasonable to combine PSA and CCL2 as biomarkers to improve the prediction of prognosis of patients with prostate cancer. As we expected, our study aiming to develop a novel risk classification showed that combining PSA and CCL2 predicted OS, PCaSS, and CFS of all patients with prostate cancer and those treated with ADT compared with PSA or CCL2 alone (Figure 6). Because PSA and CCL2 are secreted proteins present in blood, the assays of these two proteins are very easy to perform and are relatively inexpensive and less invasive.

One may raise the question that the small sample size in the present study prevented the determination of statistical significance of differences between the groups. We believe that larger prospective studies that include patients with diverse ethnic backgrounds and longer follow-up periods are required to confirm our findings. Because patients in the present study were screened for PSA before biopsy, it is unclear whether CCL2 with or without PSA will serve as a reliable biomarker for initial screening. Moreover, treatment with recently developed anti- prostate cancer agents such as abiraterone, enzalutamide, and cabazitaxel prolong the survival of patients with prostate cancer [28]. It remains to be determined if changes in CCL2 levels as well as any association of this change with the PSA level will be detected in patients with prostate cancer before or after receiving such therapy. The concentration of CCL2 used in migration assay (Figure 1A) was higher than that obtained from medium of mig cells (Figure 3A) and serum CCL2 concentration. We cannot rule out the possibility that mig cells may consume CCL2 for cell survival. Similarly, systemic CCL2 concentration may be diluted from systemic circulation after released from prostate tissue. Therefore, we think the concentration of CCL2 used in migration assay may be reasonable as the concentration in prostate tissue since previous studies have used similar concentration of CCL2 for migration assay [29, 30].

## CONCLUSION

To our knowledge, the present study is the first to investigate serum CCL2 levels using a cohort of prostate cancer samples with supporting data from *in vitro* models that reveals the predictive value of serum CCL2 levels as a biomarker of prostate cancer. The novel classification using CCL2 and PSA levels together may serve as a useful tool to improve prediction of survival and the efficacy of ADT in patients with prostate cancer.

## MATERIALS AND METHODS

### *In vitro* experiments

C4-2 cells were maintained in RPMI-1640 medium with 5% fetal bovine serum and 1% penicillin/streptomycin in a humidified 5% CO_2_ environment at 37°C. For WB analysis, cells were lysed in RIPA buffer (50 mM Tris-HCl/pH 7.4, 1% NP-40, 150 mM NaCl, 1 mM EDTA, 1 mM PMSF, 1 mM Na_3_VO_4_, 1 mM NaF, 1 mM okadaic acid, and 1 mg/ml aprotinin, leupeptin and pepstatin). Individual samples (15 μg-30 μg protein) were prepared for electrophoresis through an 8%-12% gradient SDS/PAGE gel and then transferred onto PVDF membranes (Millipore). After blocking the membranes with 5% fat-free milk in TBST (50 mM Tris, pH 7.5, containing 0.15 M NaCl and 0.05% Tween-20) for 1 h at room temperature, the membrane was incubated with appropriate dilutions of specific primary antibodies overnight at 4°C. After washing, the blots were incubated with anti-rabbit, anti-mouse, or anti-goat IgG horseradish peroxidases for 1 h. The blots were developed in ECL mixture (Thermo Fisher Scientific). Anti-tubulin, anti-GAPDH, and anti-AR antibodies were purchased from Santa Cruz Biotechnology, Inc. The anti-E-cadherin antibody was from R&D Systems. Anti-tSTAT3 and anti-pSTAT3 were from Cell Signaling Technology. Anti-MMP9, anti-Snail, and anti-N-cadherin antibodies were from Abcam. Human recombinant CCL2 was obtained from R&D systems.

For cell migration assays, 24-well transwell inserts were used according to the manufacturer's instructions, and C4-2 cells (10^5^ cells per well) were seeded in the upper chamber. Cells were incubated for 24 h. We stained cells that migrated to the lower part of the membrane and counted six random fields. The culture supernatant was used for detection of CCL2 using a human CCL2 ELISA kit (R&D Systems) according to the manufacturer's instructions.

For quantitative real-time PCR analysis (qRT-PCR), total RNA was isolated using Trizol reagent (Invitrogen) according to the manufacturer's instructions. One microgram of total RNA was subjected to reverse transcription using Superscript III transcriptase (Invitrogen). Primers used were as follows: AR forward, 5′-TATCCTGGTGGAGTTGTG-3′ and AR reverse, 5′-CAGAGTCATCCCTGCTTC-3′; TGF-β1 forward, 5′-TGCTAATGGTGGAAACCCAC-3′ and TGF-β1 reverse, 5′-ATCGCCAGGAATTGTTGCTG-3′; β-actin forward, 5′-TGTGCCCATCTAGGAGGGGTATGC-3′ and β-actin reverse, 5′-GGTACATGGTGGTGGCGCCAGACA-3′. qRT-PCR was conducted using a Bio-Rad CFX96 system with SYBR green to determine the level of mRNA expression of a gene of interest. Expression levels were normalized to that of β-actin RNA.

### Patients

Serum samples were obtained from men who underwent prostate biopsy at Kanazawa University Hospital between 2007 and 2013. We performed 10-core needle transrectal ultrasound-guided biopsy of the prostate gland using an 18 G needle, and samples were obtained from the apex, middle, base, and two outer lateral areas of bilateral peripheral zones (Supplementary Figure S7). Serum values of each biomarker were measured using commercially available kits according to the suppliers' instruction manuals: PSA (Beckman Coulter), CCL2 (R&D Systems), total testosterone (Roche), and TGF-β1 (R&D systems). Patient characteristics and survival data were obtained from their charts and retrospectively compared between patients with and without prostate cancer and compared among groups divided by each cutoff value of each biomarker. Studies were performed after receiving approval from the Institutional Review Board of Kanazawa University.

### Definitions

ADT includes surgical castration, monotherapy using a luteinizing hormone-releasing hormone (LH-RH) analog or antagonist, and combined therapy using anti-androgens and an LH-RH analog or antagonist. CRPC was defined as the status of at least three consecutive elevations of PSA level or a change of antiandrogen because of disease progression.

### Statistical analysis

The Kaplan-Meier method was used to display survival data. We determined *p* values using paired and unpaired Student *t* tests and Fisher's exact test, chi-square test for trends, the log*-*rank test to determine survival distributions, and *p* < 0.05 was considered statistically significant. Analyses were performed using Prism 5 Software (GraphPad).

## SUPPLEMENTARY FIGURES AND TABLES


