# Supplementary material for: Serum chemokine (CC motif) ligand 2 level as a diagnostic, predictive, and prognostic biomarker for prostate cancer
Source: Oncotarget. 2015 Dec 19;7(7):8389–98. doi: 10.18632/oncotarget.6690 (PMC4885000; doi:10.18632/oncotarget.6690)
Supplement: Supplementary file 1 [file oncotarget-07-8389-s001.pdf]

Serum chemokine (CC motif) ligand 2 level as a diagnostic, predictive, and prognostic biomarker for prostate cancer

Supplementary Material

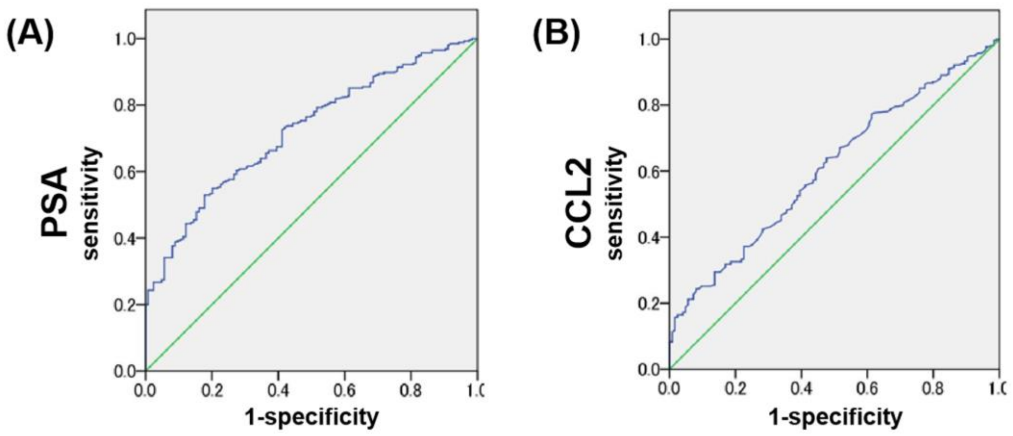

Supplementary Fig. S1 – ROC curves of PSA (A) and CCL2 (B) in 379 men who underwent prostate biopsy.

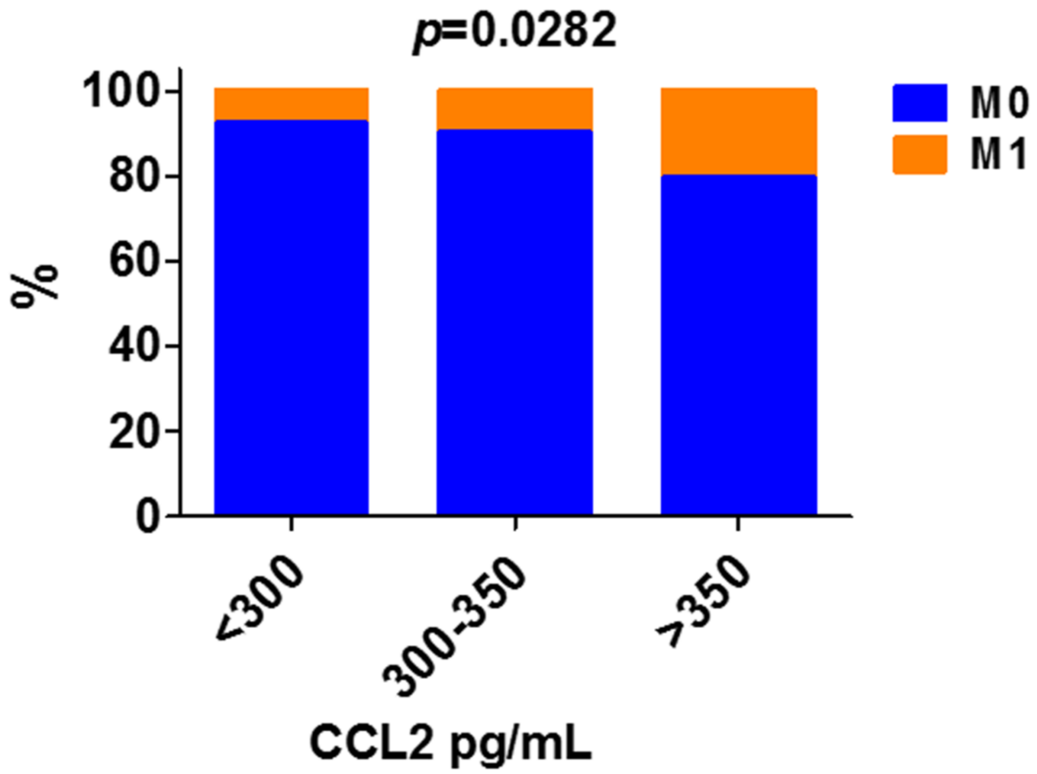

Supplementary Fig. S2 – Analysis of M1 distribution based on CCL2 levels showed that patients with higher CCL2 levels had significantly higher prevalence of meastasis (*p*=0.0282).

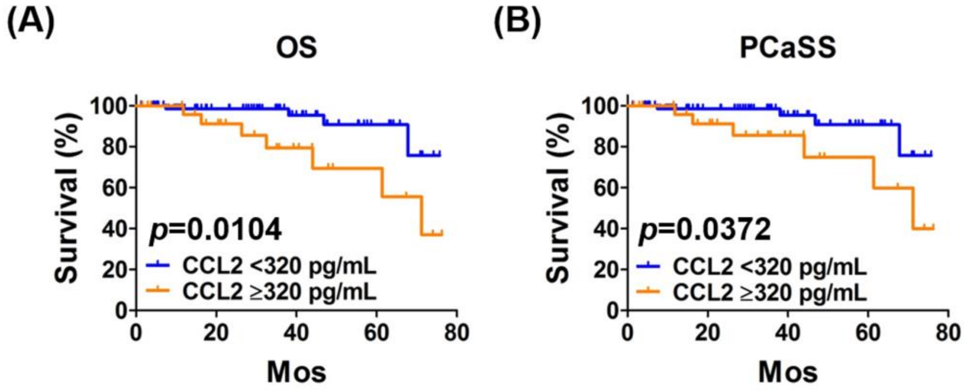

Supplementary Fig. S3 – Kaplan-Meier curves of OS (A) and PCaSS (B) in 102 patients treated with ADT were drawn and patients with CCL2 ≥320 pg/mL and CCL2 <320 pg/mL were statistically compared using log-rank test. Five-year OS rate was 68.7% and 90.8% in patients with CCL2 ≥320 pg/mL and <320 pg/mL, respectively. Five-year PCaSS rate was 74.7% and 90.8% in patients with CCL2 ≥320 pg/mL and <320 pg/mL, respectively.

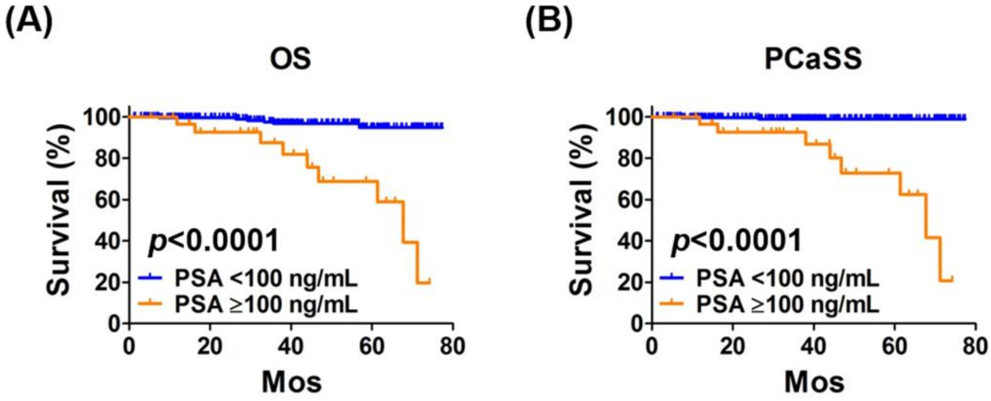

Supplementary Fig. S4 – Kaplan-Meier curves of OS (A) and PCaSS (B) in 255 patients with PSA ≥100 ng/mL and PSA <100 ng/mL were drawn and statistically compared using log-rank test. Five-year OS rate was 68.8% and 94.8% in patients with PSA ≥100 ng/mL and PSA <100 ng/mL, respectively. Five-year PCaSS rate was 72.8% and 98.9% in patients with PSA ≥100 and PSA <100 ng/mL, respectively.

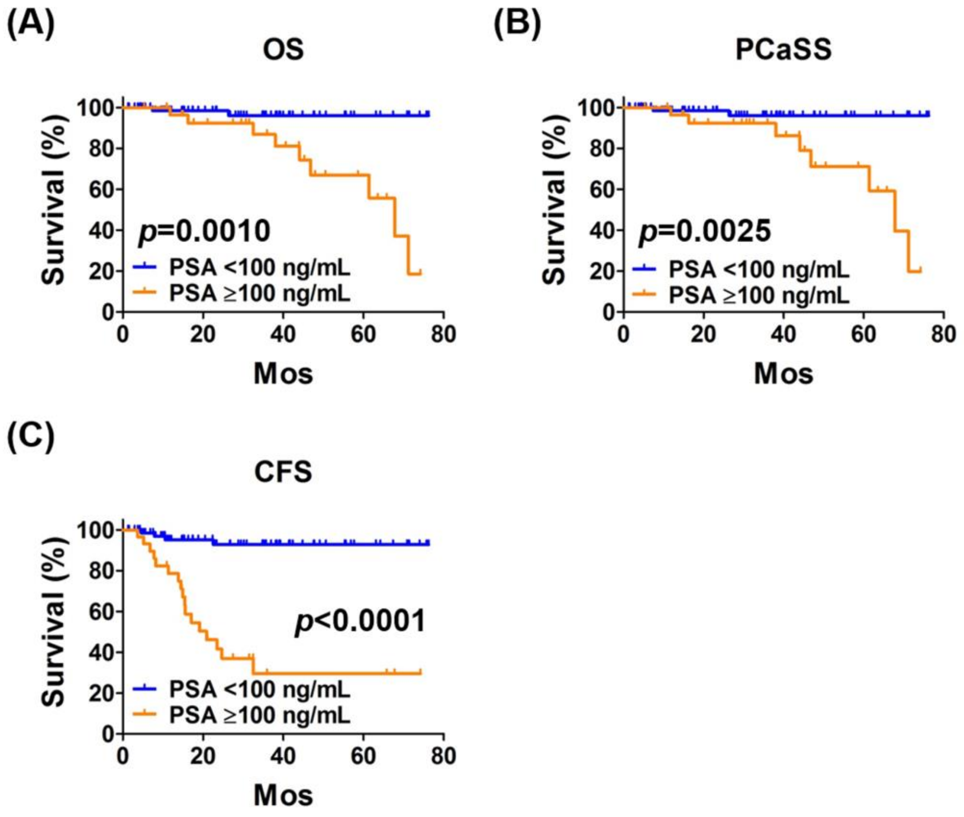

Supplementary Fig. S5 – Kaplan-Meier curves of OS (A), PCaSS (B), and CFS (C) in 102 patients treated with ADT were drawn and patients with PSA ≥100 ng/mL and PSA <100 ng/mL were statistically compared using log-rank test. Five-year OS rate was 66.9% and 96.0% in patients with PSA ≥100 ng/mL and PSA <100 ng/mL, respectively. Five-year PCaSS rate was 71.1% and 96.0% in patients with PSA ≥100 ng/mL and PSA <100 ng/mL, respectively. Five-year CFS rate was 29.6% and 92.8% in patients with PSA ≥100 ng/mL and PSA <100 ng/mL, respectively.

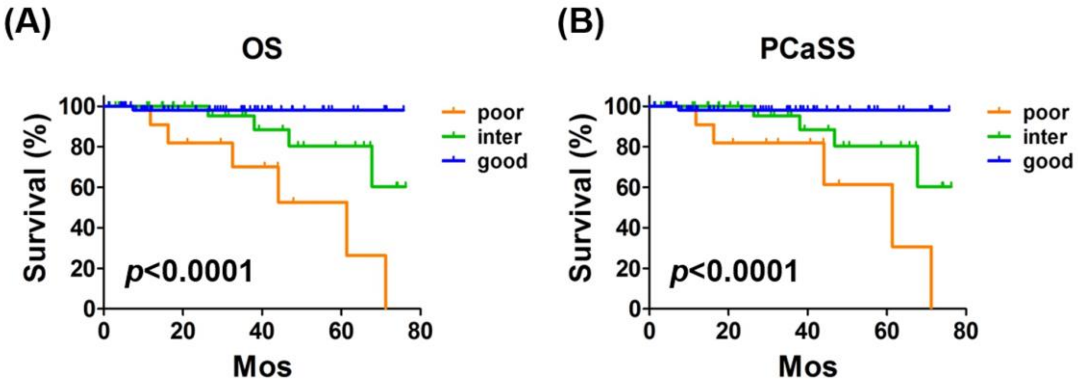

Supplementary Fig. S6 – Kaplan-Meier curves of OS (A) and PCaSS (B) in102 patients treated with ADT were drawn and patients with poor, intermediate, and good risk were statistically compared using log-rank test for trend. Five-year OS rate was 52.6%, 80.4%, and 98.0% in patients with poor, intermediate, and good risk, respectively. Five-year PCaSS rate was 61.4%, 80.4%, and 98.0% in patients with poor, intermediate, and good risk, respectively.

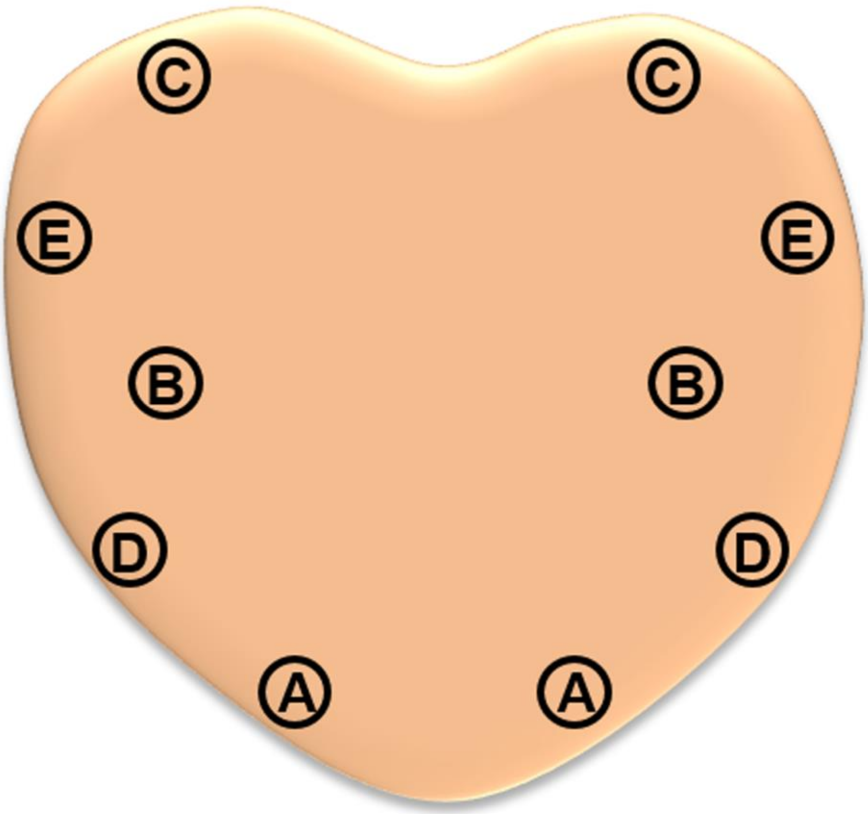

Supplementary Fig. S7 – The sites of 10-core needle transrectal ultrasound-guided biopsy of prostate gland. The apex (A), middle (B), base (C), and two far lateral areas (D, E) of bilateral peripheral zones.

Supplementary Table S1. CCL2 of biopsied patients in various PSA ranges

PSA, ng/mL		No malignancy	PCa	*p*
<41	*n*	124	204	
	Median CCL2, pg/mL	224 (70.5-394)	246 (95.3-749)	0.0005
<20	*n*	117	184	
	Median CCL2, pg/mL	224 (70.5-394)	249 (95.3-749)	0.0008
<10	*n*	100	120	
	Median CCL2, pg/mL	220 (115-394)	240 (95.3-749)	0.0043
4< <41	*n*	107	194	
	Median CCL2, pg/mL	224 (70.5-394)	247 (95.3-749)	0.0008
4< <20	*n*	100	174	
	Median CCL2, pg/mL	225.5 (70.5-394)	251.5 (95.3-749)	0.0013
4< <10	*n*	83	110	
	Median CCL2, pg/mL	223 (140-394)	243.5 (95.3-749)	0.0052

PCa = prostate cancer, PSA = prostate-specific antigen. Values in parentheses indicate range.

Supplementary Table S2. PCa patients background according to CCL2

			Overall	CCL2 ≥320 pg/mL	CCL2 <320 pg/mL	*p*
Total, *n*			255	45	210	
Median age, yr			69 (46-89)	71 (52-83)	69 (46-89)	0.1565
Median PSA, ng/mL			10.6 (1.5-16702)	13.1 (3.3-16702)	10.3 (1.5-3492)	0.1422
T, *n*	1		61	7	54	0.0021
	2		132	21	111	
	3		47	9	38	
	4		15	8	7	
N, *n*	0		228	34	194	0.0024
	1		27	11	16	
M, *n*	0		230	36	194	0.0154
	1b		17	6	11	
	1c		8	3	5	
GS, *n*	6		59	7	52	0.0027
	7		98	13	85	
	8		53	10	43	
	9		37	12	25	
	10		8	3	5	
Treatment, *n*	cADT†		78	22	56	0.0784‡
		+HDR	11	3	8	
		+seed	4	1	3	
		+EBRT	7	1	6	
		+RP	2	0	2	
	tADT		10	0	10	
		+HDR	47	5	42	
		+seed	32	7	25	
		+EBRT	7	0	7	
		+RP	16	1	15	
	HDR		2	0	2	
	Seed		17	4	13	
	RP		17	1	16	
	AS		5	0	5	

PCa = prostate cancer, GS = Gleason score, cADT = continuous androgen-deprivation therapy, HDR = high-dose-rate brachytherapy, EBRT = external beam radiotherapy, tADT = temporary ADT, RP = radical prostatectomy, AS = active surveillance. †One hundred were combined androgen blockade and 2 were monotherapy. ‡Treatments are divided into 3 groups; ADT alone, ADT+local therapy, and local therapy alone.

Supplementary Table S3. PCa patients background according to PSA

			Overall	PSA ≥100 ng/mL	PSA <100 pg/mL	*p*
Total, *n*			255	30	225	
Median age, yr			69 (46-89)	72.5 (46-86)	69 (50-89)	0.2371
Median CCL2, pg/mL			246 (95.3-749)	256.5 (165-593)	246 (95.3-749)	0.067
T, *n*	1		61	1	60	< 0.0001
	2		132	4	128	
	3		47	15	32	
	4		15	10	5	
N, *n*	0		228	11	217	< 0.0001
	1		27	19	8	
M, *n*	0		230	11	219	< 0.0001
	1b		17	13	4	
	1c		8	6	2	
GS, *n*	6		59	0	59	< 0.0001
	7		98	2	96	
	8		53	11	42	
	9		37	14	23	
	10		8	3	5	
Treatment, *n*	cADT†		78	25	53	0.0064‡
		+HDR	11	1	10	
		+seed	4	0	4	
		+EBRT	7	3	4	
		+RP	2	0	2	
	tADT		10	0	10	
		+HDR	47	1	46	
		+seed	32	0	32	
		+EBRT	7	0	7	
		+RP	16	0	16	
	HDR		2	0	2	
	Seed		17	0	17	
	RP		17	0	17	
	AS		5	0	5	

PCa = prostate cancer, GS = Gleason score, cADT = continuous androgen-deprivation therapy, HDR = high-dose-rate brachytherapy, EBRT = external beam radiotherapy, tADT = temporary ADT, RP = radical prostatectomy, AS = active surveillance. †One hundred were combined androgen blockade and 2 were monotherapy. ‡Treatments are divided into 3 groups; ADT alone, ADT+local therapy, and local therapy alone
